# Intraoperative high-resolution esophageal manometry during peroral endoscopic myotomy

**DOI:** 10.1038/s41598-020-71136-1

**Published:** 2020-08-26

**Authors:** Maximilien Barret, Marie-Anne Guillaumot, Chloé Leandri, Sarah Leblanc, Romain Coriat, Arthur Belle, Stanislas Chaussade

**Affiliations:** Gastroenterology Department, Cochin Hospital, Assistance Publique-Hôpitaux de Paris and University of Paris, 27 rue du Faubourg St Jacques, 75014 Paris, France

**Keywords:** Gastrointestinal diseases, Oesophagogastroscopy

## Abstract

Peroral endoscopic myotomy is an accepted treatment of achalasia. Some of the treatment failures can be attributable to an insufficient length of the myotomy on the gastric side, because of a more technically challenging submucosal dissection. We assessed the feasibility and the impact of an intraoperative esophageal manometry during the peroral endoscopic myotomy procedure. A high-resolution manometry catheter was introduced through the nostril before the endoscope, and left in place during the peroral endoscopic myotomy procedure. The lower esophageal sphincter pressure was recorded throughout the peroral endoscopic myotomy. The myotomy was extended on the gastric side until the lower esophageal sphincter pressure dropped below 10 mmHg. We included 10 patients (mean age = 55 years old, 3 men) treated by peroral endoscopic myotomy for type I (3/10), type II (3/10), type III achalasia (3/10) or esophagogastric junction outflow obstruction (1/10). Manometric recording was possible in all patients. The median (IQR) lower esophageal sphincter resting pressure was 23 (17–37) mmHg before myotomy, 15 (13–19) mmHg at the end of the tunnel, and 7 (6–11) mmHg at the end of the myotomy. In 4 patients out of 10, the myotomy was extended on the base of the intraoperative manometry findings. High-resolution esophageal manometry is feasible during the peroral endoscopic myotomy procedure, and leads to increase the length of the gastric myotomy in 4 out of 10 patients. However, the cumbersome nature of intraoperative high-resolution manometry during peroral endoscopic myotomy and the high frequency of gastro-esophageal reflux disease after extended gastric myotomy suggest to limit this technique to selected patients refractory to a first myotomy.

## Introduction

Peroral endoscopic myotomy (POEM) has emerged during the last decade as a major treatment of achalasia and other obstructive esophageal motility disorders^1,2^. However, primary failure occurs in up to 10% of cases^3,4^. A learning curve effect or the presence of submucosal fibrosis attributable to previous treatments have been advocated to explain these failures^3,5^. We hypothesized that a part of these failures could be explained by an insufficient length of the gastric myotomy. This can be explained by a more challenging dissection during the tunnel step at and below the gastroesophageal junction, where the orientation of the muscle changes and large vessels are more frequent. Therefore, we performed a feasibility study of an intraoperative high-resolution esophageal manometry (HRM) performed during POEM, in order to monitor the lower esophageal sphincter (LES) pressure throughout the procedure, and extend the myotomy based on the HRM findings.

## Patients and methods

This was a single center prospective study conducted at a tertiary referral center for therapeutic endoscopy and esophageal motility disorders. Ten consecutive patients with manometrically proven achalasia or esophagogastric junction outflow obstruction syndrome referred for POEM were included in the study. All patients had a pre and post POEM manometry using a tridimensional high-resolution esophageal manometry catheter and station (ManoScanTM ESO 3D and A300 Manoscan station Medtronic, Minneapolis, Mn). All patients provided written informed consent for the procedure and the use of their data. The study protocol conforms to the ethical guidelines of the 1975 Declaration of Helsinki as reflected in a priori approval by the institution's human research committee (The Ethical Review Committee for publications of the Cochin University Hospital (CLEP) : n°AAA-2018-08008).

**Figure 1:**
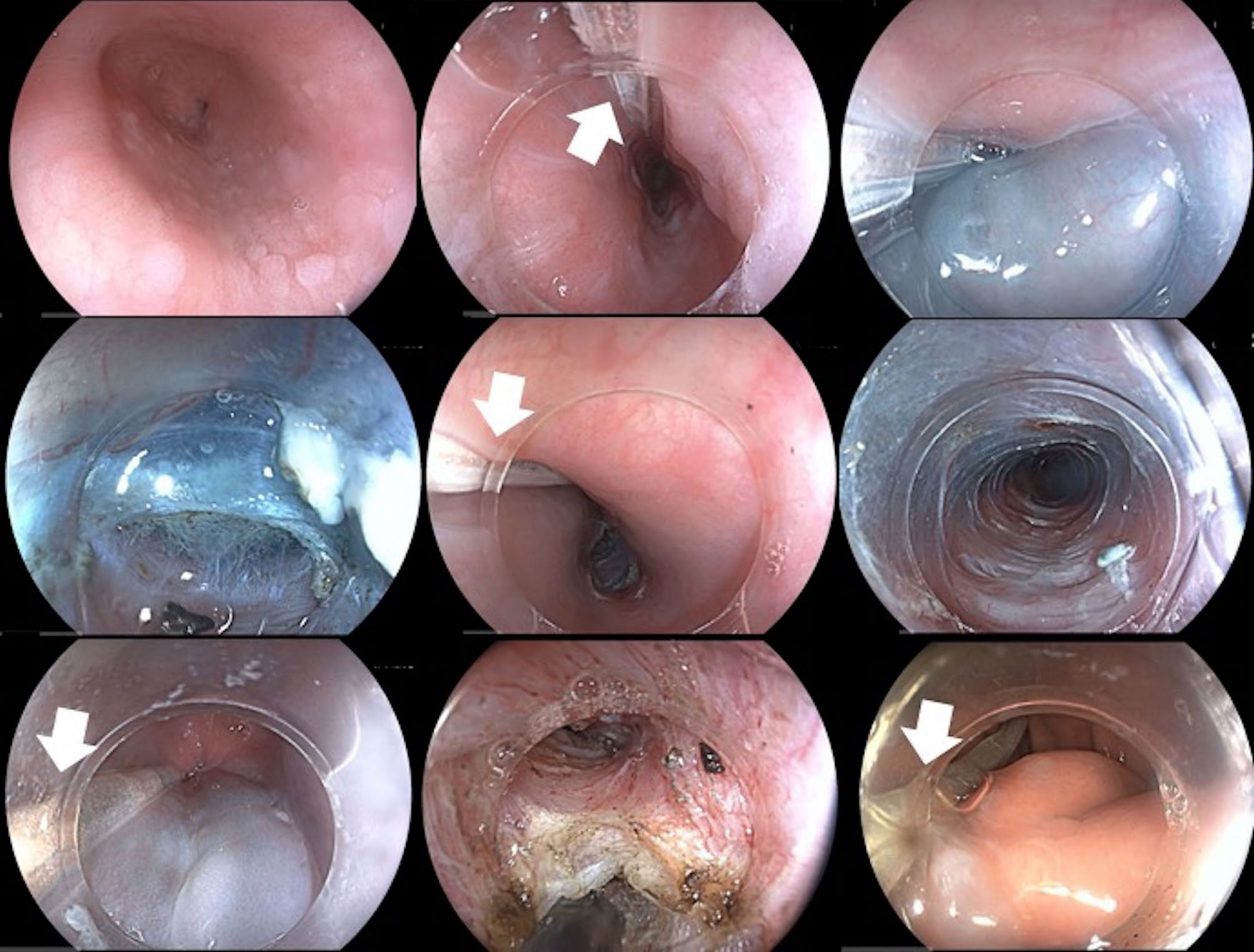
Endoscopic steps of the peroral endoscopic myotomy. Note the presence of the manometry catheter in the esophageal lumen during the whole procedure.

With patients under general anesthesia with endotracheal intubation, upper gastrointestinal endoscopy was performed to clean the esophagus. A 3D high-resolution esophageal manometry catheter with a disposable protective sheath connected to the manometry station brought to the endoscopy suite, was placed through the nostril in the esophagus and stomach. The crossing of the esophagogastric junction was ascertained by manometry, and helped if needed by the endoscope. The POEM procedure was conducted as reported by Inoue et al.^1^, and performed by an experienced operator with over 50 POEM performed (Fig. 1). The anterior (2 O’Clock) or posterior (5 O’Clock) route for the myotomy was chosen according to the maximal pressure zone on the 3D high-resolution manometry. During the procedure, landmarks were placed on the manometry recording and the LES pressure was recorded at baseline, at the end of the tunnel step, and at the end of the myotomy. We extended the myotomy further on the gastric side until the LES pressure dropped below 10 mmHg. Then, the manometry catheter was pulled out and the mucosal incision was ultimately closed with hemoclips.

The demographic characteristics, history of esophageal symptoms, previous treatments, pretreatment Eckardt score, pretreatment LES pressure, 4s-integrated relaxation pressure and manometric diagnosis according to the Chicago classification v3.0 were recorded. Procedural data including anterior or posterior myotomy, myotomy length, duration of the procedure, and LES pressure assessed during the procedure at baseline, end of the tunnel, end of the myotomy and after completion of the myotomy when needed. Symptoms of gastro-esophageal reflux and proton pump inhibitor prescription, follow-up endoscopy and 24 h pH measurement were recorded.

Baseline characteristics and outcomes were described as numbers and percentages for dichotomous variables, or as means and standard deviations or medians and interquartile ranges (IQR) for continuous variables. Differences in baseline characteristics and clinical response to treatment were determined by paired samples t-test. Correlation were studied using the Pearson correlation coefficient. Statistical analysis was performed using Graphpad software (La Jolla, Ca, USA).

## Results

10 patients, among whom 3 men, with a median (range) age of 55 (21–87) years, were included in the study. Type I, II and III achalasia were seen in 3 patients each and esophagogastric outflow obstruction syndrome in one patient. Their detailed characteristics are presented in Table 1. Six patients were treatment naïve, and among the 4 others, 3 had pneumatic dilatation, 1 botulinum toxin injection, and 1 Heller myotomy with Dor fundoplication. In two cases, EGD was needed to help pushing the HRM catheter through the EGJ to the stomach.

**Table 1:** Patient characteristics.

Male/female	3/7
Age—median (range)	55 (21–87)
Esophageal motility disorder—n	
Type I achalasia	3
Type II achalasia	3
Type III achalasia	3
EJOOS	1
Previous treatment for achalasia—n	
None	6
Botox	1
Pneumatic dilatation	4
Heller myotomy	1
Pre-POEM Eckardt score—median (IQR)	
Dysphagia	3 (2–3)
Regurgitations	2 (2–3)
Weight loss	1 (0.75–2.25)
Chest pain	0 (0–2.25)
Basal IRP, mmHg—median (IQR)	20.1 (14–33)
LES resting pressure , mmHg—median (IQR)	23.4 (21–43)

*EGJOOS* esophagogastric junction outflow obstruction syndrome, *IQR* interquartile range, *IRP* integrated relaxation pressure, *LES* lower esophageal sphincter.

**Figure 2:**
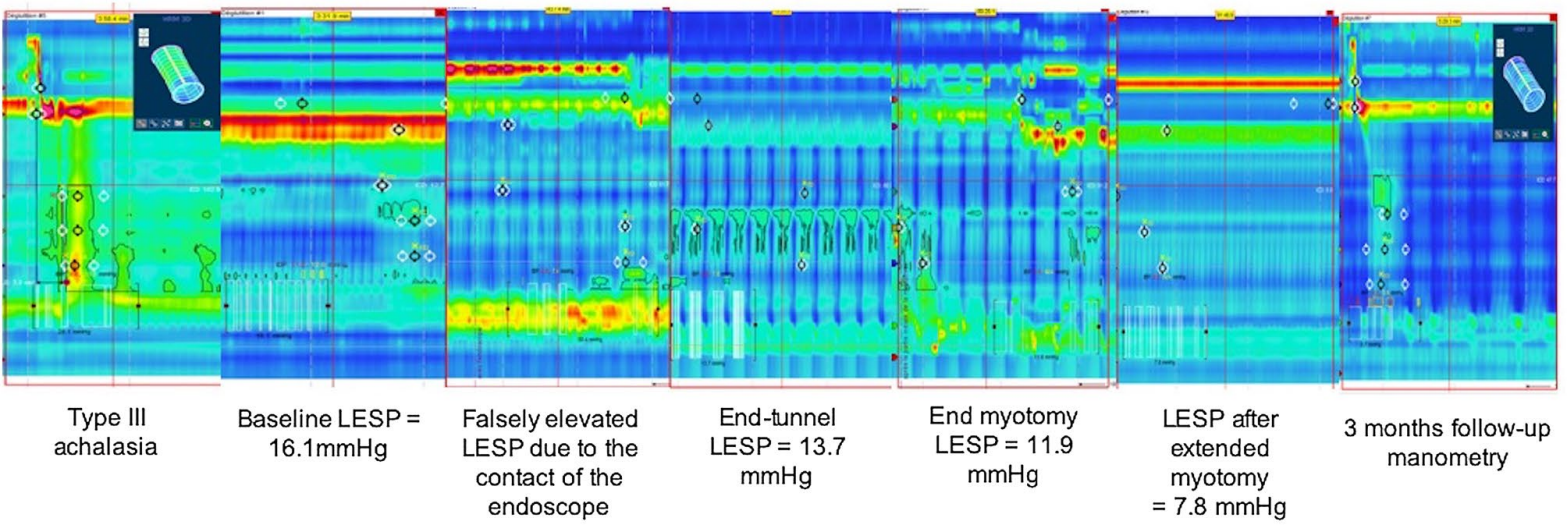
High-resolution esophageal manometry measurements before, during and after the peroral endoscopic myotomy procedure.

POEM procedures were performed in a median (IQR) of 115 (98–120) mn, and the LES pressure dropped significantly during at each step, from 23 (17–37) mmHg before the mucosal incision, to 15 (13–19) mmHg at the end of the tunnel, to 7 (6–11) mmHg at the end of the myotomy (*p* = 0.0003) as illustrated in Fig. 2. The correlation between LES mean resting pressure at HRM and intraoperative HRM is presented in Table 2. The mean ± SD pressure drop after finishing the tunnel was 9.7 ± 8 mmHg, corresponding to a mean ± SD = 32.9 ± 20% of the LES pressure drop obtained during the entire POEM procedure. The mean pressure drop after the tunnel was 13.8 ± 9 mmHg versus 3.6 ± 3 mmHg, in the treatment naïve and previously treated patient groups, *p* = 0.06. This corresponds to a 42.3% mean pressure drop in the treatment naïve versus 18.8% in the previously treated group (*p* = 0.06). POEM and HRM recordings were feasible in all cases, without differences between anterior and posterior myotomies. No adverse event was attributable to the per POEM manometry, and no damage to the manometry catheter occurred. In 4 patients, the gastric myotomy was extended on the basis of the per POEM manometry (LES pressure remaining above 10 mmHg after the initial myotomy) of a median (IQR) 2.5 (2–3.75) cm. Of these four patients, there was one patient of each achalasia subtype and one patient with esophagogastric junction outflow obstruction. Half of them (2/4) had been previously treated for achalasia. Procedural data is presented in Table 3.

**Table 2:** Lower esophageal sphincter pressure during conventional and intraoperative high-resolution manometry.

Patient	Mean LES resting pressure during HRM (mmHg)	LES pressure during intraoperative (sedated) HRM (mmHg)	Pearson’s correlation
1	22.7	43	r = 0.56
2	62.6	22.3	
3	36.9	16.1	
4	113.2	48	
5	24	17.4	
6	25.3	21.8	
7	11.6	11.1	
8	21.4	35.1	
9	20.7	23.3	
10	20.9	22.8	

*HRM* high-resolution manometry, *LES* lower esophageal sphincter.

**Table 3:** Procedural characteristics of the peroral endoscopic myotomy and results of the intraoperative high-resolution manometry (n = 10).

POEM duration, mn—median (IQR)	115 (98–120)
Anterior/posterior myotomy	2/8
Length of the myotomy, cm—median ( IQR)	12 (8–14)
Per POEM HRM	
Initial LES pressure, mmHg—median (IQR)	23 (17–37)
End-tunnel LES pressure, mmHg—median (IQR)	15 (13–19)
Final LES pressure, mmHg—median (IQR)	7 (6–11)
Modification in the length of the myotomy	N = 4
Initial length of the myotomy (cm)	9/10/11/13
Final length of the myotomy (cm)	12/12/13/17

*POEM* peroral endoscopic myotomy,* IQR* interquartile range.

Two mucosotomies were observed during the POEM procedure, closed by hemoclips, without esophageal leak on the esophagram performed at day one before resuming oral food, as per standard procedures in our department. No early postoperative complication was observed. After a mean follow-up of 28 ± 3 months, no treatment failure was observed. The median (IQR) Eckardt score changed from 7.5 (5–9) before treatment to 1 (0–3) after treatment (*p* < 0.0001). Clinical gastro-esophageal reflux symptoms was observed in half of the patients and three out the four patients with an extended myotomy (*p* = 0.5). The mean acid exposure time was 16.6% versus 12.4%, *p* = 0.4 for the extended versus conventional myotomy patient groups, respectively. The outcomes after POEM are presented on Table 4.

**Table 4:** Patient outcomes after POEM.

Post-POEM Eckardt score—median (IQR)	
Dysphagia	1 (0–1)
Regurgitations	0 (0–2)
Weight loss	0 (0–0)
Chest pain	0 (0–1)
Basal IRP, mmHg—median (IQR)	6.4 (4–10)
LES resting pressure, mmHg—median (IQR)	9.8 (8–17)
Gastro-esophageal reflux	
Clinical symptoms	5/10
Erosive esophagitis	3/10
Abnormal esophageal acid exposure	4/10

*IQR* interquartile range, *IRP* integrated relaxation pressure.

## Discussion

Our work suggests the feasibility and safety of a high-resolution manometry performed during the POEM procedure. The intraoperative manometry changed the patient management in 4/10 cases, leading us to extend the gastric myotomy due to a persistently elevated LES pressure.

To our knowledge, this is the first study to report on the feasibility of intraoperative manometry during POEM. Several teams have searched to tailor the treatment of achalasia with intraprocedural measures of the LES pressure and relaxation. Del Genio et al. used intraoperative esophageal manometry to enable a calibration of the myotomy and of the fundoplication during laparoscopic Heller myotomy^6^. Based on 144 patients with achalasia, they identified a residual high-pressure zone requiring an extension of the myotomy in 15% of patients. Furthermore, this team achieved calibrated Nissen fundoplications after Heller myotomies, allowing for an optimal reflux control and similar symptom scores when compared to Heller myotomy and conventional Dor fundoplication. Of note, these results have also been reported by other teams, although on smaller patient numbers^7^. Esophagogastric junction distensibility measurement using the EndoFLIP (Crospon, Galway, Ireland) system was also reported during POEM by two groups : Familiari et al. documented the increase of the esophagogastric junction distensibility after POEM using a 30 mL balloon, however with little impact on the procedure itself^8^. Conversely, Teitelbaum et al., using a 40 mL balloon, observed an increase in the esophagogastric junction distensibility after the submucosal tunneling step, and after section of the lower esophageal sphincter, without improvement after extension of the gastric myotomy over 3 cm^9^. A recent review of the 6 published studies in the field concluded that the final intraoperative esophagogastric junction distensibility measurements did not differ significantly between good and poor responders to POEM^10^. Finally, this system was also used intraoperatively after pneumatic dilatation for achalasia, and proved useful to predict immediate clinical response, and guide an increase in the dilator size within a single endoscopy session^11^. Overall, intraoperative EndoFLIP might to be an interesting alternative to intraoperative HRM to help determining the distal extension of the myotomy, with the advantage of a single use catheter with less consequences in case of damage by the endoscope or the endoknife, but potential safety concerns in case of repeated manoeuvers of this large balloon catheter through the esophagogastric junction during POEM.

Our aim was to investigate the potential of per procedure HRM to guide the myotomy on the gastric side. Indeed, the tunnel procedure at the level of the cardia, particularly in case of prior therapy (as in 4/10 of our patients) can become more difficult. Furthermore, the intragastric part of the tunnel can be more technically challenging, in terms of orientation and hemostasis of large vessels. We hypothesized that an insufficient gastric myotomy, explained by these technical concerns, could account for the technical failures of some of the POEM procedures.

The pressure drop obtained after submucosal tunneling was an unexpected finding of our work: indeed, 33% of the LES pressure drop obtained during the POEM procedure followed the submucosal tunneling alone. Furthermore, the effect of submucosal tunneling alone on the LES pressure drop was more pronounced in pretreated patient. Of note, Teitelbaum et al., in a study measuring the esophagogastric junction distensibility during POEM, also observed a significant pressure drop after submucosal tunneling alone^9^. This suggests that submucosal changes during achalasia could play a role in the impaired relaxation of the esophagogastric junction.

A persistent elevated LES pressure above 10 mmHg has been showed in patients treated with pneumatic dilatation to be predictive of early symptom recurrence^12^. Although this cutoff value has been defined in awake patients, and the correlation between the sedated and awake HRM value of LES pressure is uncertain, we chose to aim at bringing the LES pressure below 10 mmHg in order to diminish the LES pressure as much as possible, and found that this cutoff was not met in 4/10 of our patients after what we considered to be a complete myotomy. The only specific characteristic of the patients requiring an extended gastric myotomy was the highest proportion (2/4) of previously treated patients. Prior treatment can result in a more difficult dissection at the level of the esophagogastric junction, accounting for an insufficient myotomy. This could lead us to advise intraoperative HRM in POEM for recurrent achalasia, where its clinical contribution seems to be the highest. Of note, the 4s integrated relaxation pressure (4s-IRP), the metric used during HRM to define an impaired relaxation of the esophagogastric junction, was not measurable in our patients: first, because the software did not allow us to calculate the 4s-IRP during the recording of the manometry; second, because no swallows occurred in our patients under general anesthesia. One may question the applicability of the 10 mmHg cutoff , meant to predict recurrence after pneumatic dilatation, to post POEM recurrence, all the more several large POEM studies report a mean post POEM LES > 10 mmHg and a good short term efficacy^13^. However, the few long-term data available on POEM show that despite a 91–94% short-term clinical success, the 5-year clinical success rate drops to 77–87%^14^. Therefore, it is possible that the conclusions from Eckardt et al. remain true for POEM patients, and that post POEM patients with a LES pressure > 10 mmHg with will eventually develop recurrent achalasia.

The main limitation of our study is that the measures performed under general anesthesia make most numbers hardly comparable to usual HRM figures, and probably minimize the diaphragmatic component of the LES pressure. However there was a moderate but positive correlation (r = 0.56) between the mean LES pressure at HRM and the initial LES pressure measured before the start of the POEM. Second, the presence of the HRM station in the endoscopy suite, close to the patient head, makes of per POEM HRM a cumbersome and lengthy procedure requesting the presence of a second gastroenterologist throughout the procedure. Third, the small patient number only allows us to assess the feasibility of per POEM HRM: comparative outcomes with conventional POEM would require an adequately powered study. Although intraoperative HRM could theoretically help in guiding the length of the myotomy in patients with type III achalasia, the absence of esophageal contractions under general anesthesia precludes this use of intraoperative HRM. Furthermore, the higher frequency of GERD symptoms in patients with an extended myotomy calls into question the part of the stomach where the gastric myotomy should be best extended^15^.

Finally, intraoperative esophageal HRM during POEM is feasible and safe, and might help tailoring the length of the myotomy to each patient. However, the procedure is relatively time consuming and should be limited to difficult cases, such as redo POEM.
